# Chromatic imaging properties of myopia control spectacle lenses

**DOI:** 10.1364/BOE.545932

**Published:** 2025-03-19

**Authors:** Augusto Arias, Susanna P. Clement, Pablo Artal, Siegfried Wahl

**Affiliations:** 1 ZEISS Vision Science Lab, Ophthalmic Research Institute, University of Tübingen, Tübingen, Germany; 2 Laboratorio de Óptica, Universidad de Murcia, Campus de Espinardo, Murcia 30100, Spain; 3 Carl Zeiss Vision International GmbH, Technology & Innovation, Aalen, Germany

## Abstract

Myopia progression in children can lead to ocular morbidity during adulthood. Spectacle lenses have been developed and commercialized for myopia control (MC), but their imaging properties have only been assessed under monochromatic illumination. In this study, we quantified the chromatic imaging properties (wavelengths, 450, 532 and 635 nm) of four MC lenses and a single vision lens at three retinal eccentricities (0°, 20° and 30°) along the horizontal meridian using spatial light modulation technology. Our results suggest that the design of myopia-control lenses based on simultaneous competing blurring should enhance the quality of images projected in front of the peripheral retina at long wavelengths.

## 1. Introduction

Myopia is a common refractive error in which far objects are focused in front of the retina of a relaxed eye. It is usually corrected using single-vision ophthalmic lenses; however, this approach is ineffective in preventing myopia progression, which may lead to serious ocular pathologies (e.g., myopic macular degeneration) [1]. Current strategies for myopia control (MC), aimed at slowing myopia progression, include: increasing time spent outdoors, using pharmaceutical agents, and wearing ophthalmic lenses, such as orthokeratology or multifocal contact lenses, as well as specialized spectacle lenses with incorporated lenslets or microdiffusers at their periphery. Specialized spectacle lenses are a safe option, as they mitigate the risks of infections associated with contact lens use and the potential side effects linked to pharmaceutical agents. Furthermore, this approach is unaffected by external factors that may limit outdoor activity, such as public policies or weather conditions. However, the efficacy of MC spectacle lenses in slowing the progression of ocular axial elongation or refractive error is moderate [2] and depends on the optical characteristics of the treated myopic eye [3]. A better understanding of the imaging properties of the MC-spectacle lenses and their interaction with the wearer’s ocular optics can improve the benefits of this type of treatment.

Previously, the imaging properties of MC spectacle lenses have been evaluated by retrieving the wavefront of their peripheral optical structures [4–6] or recording images through them [7,8] using monochromatic light. However, light signaling for ocular elongation or shortening is probably also influenced by chromatic cues, as investigated in animal and human models [9,10]. For example, humans showed an increase or decrease in ocular axial length after exposure to long or short wavelengths, respectively [11]. Moreover, it was suggested that the wavelength dependence of ocular refraction plays a crucial role in regulating eye growth [12]. Therefore, a comprehensive characterization of MC lenses should account for the effects of the chromatic ocular aberrations over a wide range of eccentricities.

In this work, we use a liquid crystal-spatial light modulator to characterize the chromatic dependence of the imaging properties of four spectacle lenses currently used for MC and one standard single-vision spectacle lens used as a reference. The lenses were tested alone and with aberrations of two myopic eyes. In our approach, point spread functions (PSFs) were recorded while illuminating the lenses at three different eccentricities, three visible wavelengths (450, 532 and 635 nm), and varying defocus amounts. The recorded PSFs were processed to calculate the area under the modulation transfer function (AUMTF) as an image quality descriptor. This PSF-based analysis may be more suitable for characterizing lenses with incorporated diffusers than an analysis-based wavefront measurement since the spatial resolution of the wavefront sensors is typically lower than the spatial features of the diffusers. We discuss the characterization results to analyze their implications for MC-lenses design.

## 2. Methods

### 2.1. Tested spectacle lenses

**Fig. 1.**
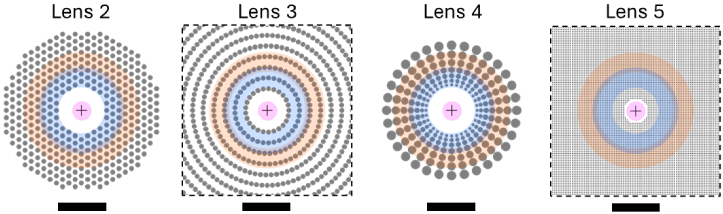
Arrangements of optical structures in the tested myopia-control lenses. The crosses mark the lenses’ centers. The magenta, blue and orange shadows depict the approximated zones where the lenses were illuminated at eccentricities of 0°, 20° and 30°, respectively. Scale bars, 10 mm.

Five spectacle lenses were tested: 1. a single-vision lens (Carl Zeiss Vision International GmbH, Germany); 2. a MiYOSMART (Hoya Corporation, Japan); 3. a Stellest (Essilor International, France); 4. a MYOpis Boosted (Divel Italia, Italy); 4. a SightGlass (CooperVision, United States of America). The nominal base power of all the lenses was -2 D. Other characteristics of the tested lenses are listed in Table 1. Lenses 2 to 5 were designed for myopia-control, presenting optical structures on the periphery of a single-vision base lens that corrects the central refraction. The arrangements of these structures are sketched in Fig. 1.

**Table 1.** Characteristics of the tested lenses. $V_{d}$ , Abbe number from [13]. ε , eccentricity of the illumination.

	Material	Structures in the periphery			Refs
		Optical elements	Type of arrangement	Fill factor^*a*^	
Lens 1	CR39 (*V_d_*, 58)	×	×	×	×
Lens 2	Polycarbonate (*V_d_*, 30)	Spherical lenslets (size, 1.03 mm; power, 3.5 D)	Hexagonal (period, 1.5 mm)	40%	[4]
Lens 3	Polycarbonate (*V_d_*, 30)	Aspherical lenslets (size, 1.0 mm) with varying powers (range, 6 to 3.5 D)	11 annular rings (period, 2.3 mm)	28%	[4]
Lens 4	Polycarbonate (*V_d_*, 30)	Lenslets with varying sizes (range, 0.6 to 1.9 mm)	7 annular rings	61% at *ε*=20° 56% at *ε*=30°	[14]
Lens 5	Trivex (*V_d_*, 45)	Micro-diffusers (average size, 140 μm)	Square (period, 365 μm)	∼12%	[15]

^*a*^ Averaged fill factor.

### 2.2. Acquisition of chromatic PSFs

**Fig. 2.**
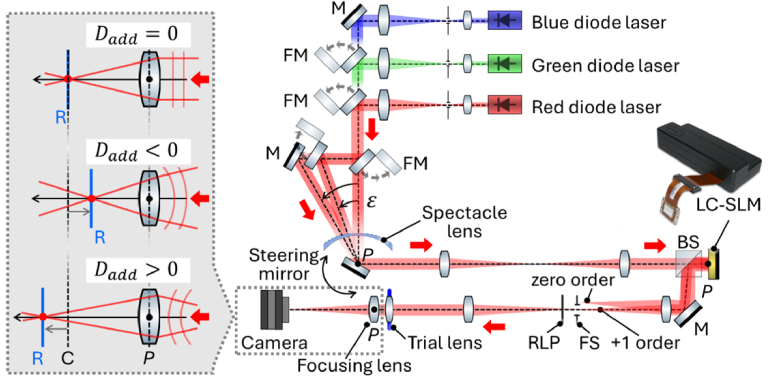
Instrument for acquiring the through-focus point spread functions of the spectacle lens at blue, green and red wavelengths and eccentricities ε . Inset, effects of programming added defocus (*D_add_*) with different signs on the SLM. The added defocus shifts the focal positions with respect to the camera sensor plane (C). M, mirror; FM, foldable mirror; BS, beam splitter; LC-SLM, liquid crystal spatial light modulator; P, pupil planes; FS, field stop; RLP, rotational linear polarizer; R, retinal plane.

Figure 2 shows the optical setup used to acquire the polychromatic PSFs of the tested spectacle lenses. This setup is based on a previously developed instrument to characterize the focusing properties of spectacle lenses at a single wavelength [7]. In the current setup, three collimated beams from three diode lasers (central wavelengths, $\lambda_{B}=450\;\text{nm}$ , $\lambda_{G}=532\;\text{nm}$ , and $\lambda_{R}=635\;\text{nm}$ ) illuminated the spectacle lens at eccentricities (*ε*) of 0°, 20° and 30°. Positive and negative *ε* values represent temporal and nasal retinal angles, respectively. After the light passes through the spectacle lens, a steering mirror guides the beams toward a phase-only, liquid crystal spatial light modulator (SLM; LETO, Holoeye Photonics AG, Germany). The mirror was optically conjugated with the SLM using a telescope with unitary magnification. A second telescope conjugated the SLM with a focusing lens that images the PSF on a monochromatic camera (DMK23UM021, The Imaging Source GmbH, Germany). The exit pupil of the instrument coincides with the second principal plane of the focusing lens. The phase modulation of the SLM was calibrated at each wavelength. A rotating linear polarizer (RLP in Fig. 1), positioned between the SLM and the focusing lens, modulates the PSF peak to prevent saturation or underexposure of the digitized PSF. This adjustment is possible because the light modulated by the SLM is horizontally polarized.

For each PSF acquisition, the phase map displayed on the SLM (*W_SLM_*) corresponds to:

(1) $$W_{SLM}{|}_{\lambda ,\varepsilon }(D_{add})=W_{grating}+W_{system}{|}_{\lambda }+W_{defocus}{|}_{\lambda }(D_{add})+W_{ocular}{|}_{\lambda ,\varepsilon }$$

Each component is described as follows. $W_{grating}$ is a binary grating (period, 12.8 μm) used to shape the pupil as an ellipse – with vertical and horizontal lengths of ∅︀ and ∅︀cos(*ε*), respectively – using the phase-only SLM. The grating depth was set to π rad within the pupil and zero otherwise. To carry out the amplitude shaping, a field-stop between the SLM and the focusing lens filters out diffraction orders other than +1. $W_{system}{|}_{\lambda }$ is the phase map that mitigates the instrument’s aberrations at each *λ*. This map is generated through a hill-climbing approach where the PSF peak is maximized by varying the coefficients of the Zernike polynomials ( $Z_{n}^{m}$ , following the OSA double index notation [16]) between the 2^nd^ and 4^th^ orders. $W_{defocus}{|}_{\lambda }$ is a spherical phase map with variable defocus $D_{add}$ at each *λ* used to acquire the through-focus PSF and calculated as $(\pi /\lambda )D_{add}r^{2}$ , where *r* is the radial coordinate at the pupil plane. The inset of Fig. 2 illustrates how programming $D_{add}$ allows registering PSFs that end up behind, in front of, and at the retina. When *D_add_*=0, the retinal plane coincides with the camera sensor plane, enabling the acquisition of PSFs at the retina. Setting a negative $D_{add}$ shifts the retinal plane axially toward the focusing lens, enabling the capture of intensity distributions that occur behind the retina. Conversely, setting a positive $D_{add}$ shifts the retinal plane axially behind the camera sensor plane, allowing the recording of intensity distributions as they occur in front of the retina. $W_{ocular}{|}_{\lambda ,\varepsilon }$ represents each eye’s ocular aberrations (OAs) at particular *λ* and *ε* values, whose calculation is detailed in the following subsection.

The procedure to acquire the PSFs is described as follows. First, a trial lens was located immediately in front of the focusing lens to compensate for the central dioptric power of the spectacle lens, preserving the best focal position on the camera sensor. Second, the instrument aberrations (including those originating from the trial lens) at each wavelength were corrected as described above. Third, the spectacle lens was positioned and aligned 20 mm in front of the steering mirror using a 3D-printed ruler mounted on this mirror. This distance corresponds to the vertex distance of 16 mm plus the distance between the cornea apex and the entrance eye’s pupil in air (∼ 4 mm). The 20 mm distance was imposed by the dimensions of the optical and mechanical components. Fourth, the beams passing through the central clear zone of the spectacle lens (i.e., at *ε*=0°) were axially shifted – using the SLM – to maximize the intensity on the camera sensor only at green wavelength. This approach mitigates the effects of variations in the base power of the tested lenses and discrepancies between the used and standard (∼13 mm) vertex distances on the focusing properties while maintaining the wavelength-dependent performance characteristics of the spectacle lenses. Finally, the PSFs were acquired for each eccentricity and wavelength.

The modulation transfer function (MTF) was computed from each PSF by applying the fast Fourier transform. The area under the MTF – up to 60 cycles per degree – (AUMTF) was calculated as a metric of image quality. The reported AUMTF was normalized to its value for the instrument corrected, without a spectacle lens, at $\lambda_{G}$ and *ε*=0°. The longitudinal chromatic aberration (LCA), corresponding to the difference in the best focal positions between $\lambda_{B}$ and $\lambda_{R}$ , was assessed in the spectacle lenses without and with reproducing the OAs. Moreover, the transverse chromatic aberration (TCA) of the spectacle lenses was measured. To visualize the effects of chromatic aberrations on the peripheral PSFs, polychromatic PSFs were generated by encoding the monochromatic red, green, and blue PSFs in their respective channels of the RGB format.

According to the simultaneous competing blur theory [17], myopia progression can be slowed by projecting sharp images in front of the peripheral retina. A single-value metric, termed mAUMTF, was computed to assess the capacity of the tested lenses in increasing the quality of such images at *ε*=±20° and ±30°. mAUMTF was defined as the integral of the AUMTF values in front of the retina (i.e., for $D_{add}$ within the range [0.1,5] D), normalized by the integral of the AUMTF values across both sides of the retina (i.e., for $D_{add}$ within the range [–5,5] D):

(2) $$\text{mAUMTF}=\frac{\sum_{D_{add=0.1}}^{5}\text{AUMTF}(D_{add})\Delta D_{add}}{\sum_{D_{add=-5}}^{5}\text{AUMTF}(D_{add})\Delta D_{add}}$$

where $\Delta D_{add}$ is the resolution of the added defocus when acquiring the through-focus PSF. $\Delta D_{add}$ was 0.1 D for |*D_add_*|≤1 D and 0.2 D otherwise.

### 2.3. Reproduction of wide-field ocular aberrations

Jaeken *et al*. [18] measured the OAs of 6 right myopic eyes (age, mean ± std: 31 ± 3 years) at 7 eccentricities (range, -30° and 30°) along the horizontal meridian using three wavelengths ( $\lambda_{Ba}=473\;\text{nm}$ , $\lambda_{G}=532\;\text{nm}$ and $\lambda_{Ra}=671\;\text{nm}$ ). These OAs were reported as the coefficients $\left(c_{i}{|}_{\varepsilon ,\;\lambda }\right)$ of the Zernike polynomials – up to the 4^th^ order – scaled to a pupil diameter (∅︀) of 4 mm. The OAs of two myopic eyes with similar central defocus errors (within the range of interest for MC), but different peripheral refractions and amounts of high-order aberrations, were reproduced to study their interaction with the tested MC lenses. The central refractions of these eyes were: Eye 1, sphere –3.25 D, cylinder -0.42 D; and Eye 2, sphere – 3.26 D, cylinder -0.29 D. A spectacle lens with a base power of -3.2 D at a standard vertex distance of 13-mm would correct the central defocus error of both eyes.

**Fig. 3.**
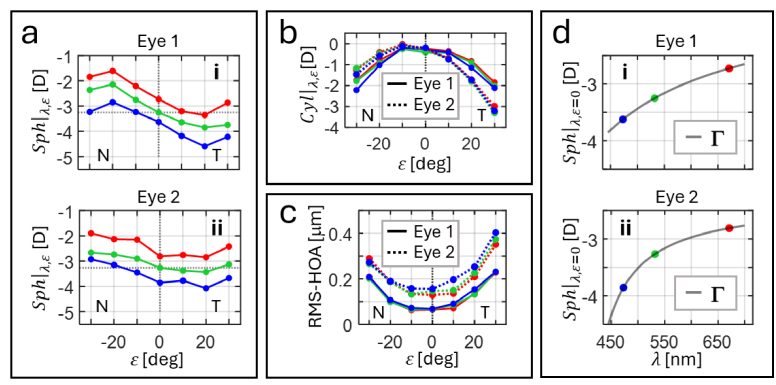
Optical characteristics of the reproduced eyes. (a) Measured spherical error across the eccentricity ε along the horizontal meridian of eye 1 (a.i) and 2 (a.ii). (b) Measured cylindrical and (c) root-mean-square of high order aberrations (RMS-HOA) as a function of ε . (d) Central spherical error as a function of the wavelength λ , which is fitted with Cornu’s hyperbolic formula Γ . T and N in (a), (b) and (c) denote the temporal and nasal range of retinal eccentricities, respectively. Red, green and blue lines and symbols depict the values for each spectral region.

The amount of spherical (Sph) and cylindrical (Cyl) errors – as a function of the eccentricity and wavelength – were calculated from the Zernike coefficients as [19]:

(3) $$Sph{|}_{\lambda ,\varepsilon }=-\frac{16\sqrt{3}}{\emptyset^{2}}C_{2}^{0}{|}_{\lambda ,\varepsilon }$$

(4) $$Cyl{|}_{\lambda ,\varepsilon }=-\frac{16\sqrt{6}}{\emptyset^{2}}\sqrt{{\left({C_{2}^{+2}|}_{\lambda ,\varepsilon }\right)}^{2}+{\left({C_{2}^{-2}|}_{\lambda ,\varepsilon }\right)}^{2}}$$

where $C_{2}^{0}{|}_{\lambda ,\;\varepsilon },\;C_{2}^{+2}{|}_{\lambda ,\;\varepsilon }$ and $C_{2}^{-2}{|}_{\lambda ,\;\varepsilon }$ are the Zernike coefficients corresponding to defocus, horizontal and oblique primary astigmatism, respectively. Figures 3(a) and 3(b) shows the $Sph{|}_{\lambda ,\varepsilon }$ and $Cyl{|}_{\lambda ,\varepsilon }$ values, respectively, for the reproduced eyes.

The amount of high-order aberrations (HOAs) was quantified by the root-mean-square of HOAs (RMS-HOA) and calculated by $\sqrt{\sum_{n=3}^{4}\sum_{m=-n}^{n}{\left({C_{n}^{m}|}_{\lambda ,\varepsilon }\right)}^{2}}$ . Figure 3(c) shows the RMS-HOA as a function of ε at each wavelength.

The computation of $W_{ocular}{|}_{\lambda ,\varepsilon }$ for these eyes involves: i. to simulate an optimal refractive correction by the base power of the spectacle lens by subtracting the 2nd order coefficients at *ε*=0° and $\lambda_{G}$ from the corresponding coefficients at other *ε* and λ values; and ii. to correct for the focal shift originated from the difference between the blue and red wavelengths of the aberrometer ( $\lambda_{Ba}$ and $\lambda_{Ra}$ ) and the current setup ( $\lambda_{B}$ and $\lambda_{R}$ ). For the latter activity, $Sph{|}_{\lambda ,\varepsilon }$ was fitted using Cornu’s hyperbolic formula (or Γ function) [20]:

(5) $$\Gamma (\lambda )=p-\frac{q}{\lambda -g}$$

where *p*, *q* and *g* are the parameters determined for each eye and eccentricity. Figure 3(d) shows Γ for each eye at *ε*=0°. Additionally, Γ and the fitting parameters for both eyes across *ε* are shown in Figure S1 and Table S1, respectively, in Supplement 1. Then, a wavelength-dependent amount B(λ) was subtracted from the Zernike coefficients for defocus at all *ε* values. B(λ) was computed as:

(6) $$B(\lambda )=\{\begin{array}{cc} \left(\emptyset^{2}/16)[\Gamma (\lambda_{B})-\Gamma (\lambda_{Ba})\right], & \lambda =\lambda_{B}\\ \left(\emptyset^{2}/16)[\Gamma (\lambda_{R})-\Gamma (\lambda_{Ra})\right], & \lambda =\;\lambda_{R}\\ 0, & \lambda =\lambda_{G} \end{array}$$

Finally, $W_{ocular}{|}_{\lambda ,\varepsilon }$ was calculated as:

(7) $$\begin{array}{ll} W_{ocular}{|}_{\lambda ,\varepsilon } & =\frac{2\pi }{\lambda }[({C_{2}^{-2}|}_{\lambda ,\varepsilon }-{C_{2}^{-2}|}_{\lambda_{G},0})Z_{2}^{-2}+({C_{2}^{0}|}_{\lambda ,\varepsilon }-{C_{2}^{0}|}_{\lambda_{G},0}-B(\lambda ))Z_{2}^{0}\\ & \;+({C_{2}^{+2}|}_{\lambda ,\varepsilon }-{C_{2}^{+2}|}_{\lambda_{G},0})Z_{2}^{+2}+\sum_{n=3}^{4}\sum_{m=-n}^{n}{C_{n}^{m}|}_{\lambda ,\varepsilon }Z_{n}^{m}] \end{array}$$

The effect of OAs on the through-focus AUMTF was evaluated at ε equals 0, ± 20° and ±30°. Nevertheless, the lenses were only illuminated at *ε*≥0. Leveraging the symmetry of the tested lenses along the horizontal meridian, the effects of OAs for *ε*<0 were evaluated by illuminating the lenses at |ε| and displaying $W_{ocular}$ corresponding to ε but horizontally flipped in the SLM.

Previously, it was noted that spectacle lenses with a base power of -3.2 D would correct the central refractive errors of the two reproduced eyes. However, the lenses being tested have a base power of -2 D. According to the lens maker's formula, a -3.2 D lens generates an LCA that is 3.2/2 times larger than that of a -2 D lens. To account for this difference, the through-focus AUMTF curves obtained with OAs for blue and red were numerically adjusted. Specifically, the defocus differences between the best focal positions for red and blue, relative to green, were sacled by 3.2/2 times their originally measured values.

Last, since the ocular TCA was not included in the aberrometry data, the ocular TCA for the spectral intervals [450,532] and [532,635] nm was calculated using Eqs. (5) and (6) in Ref. [10].

## 3. Results

**Fig. 4.**
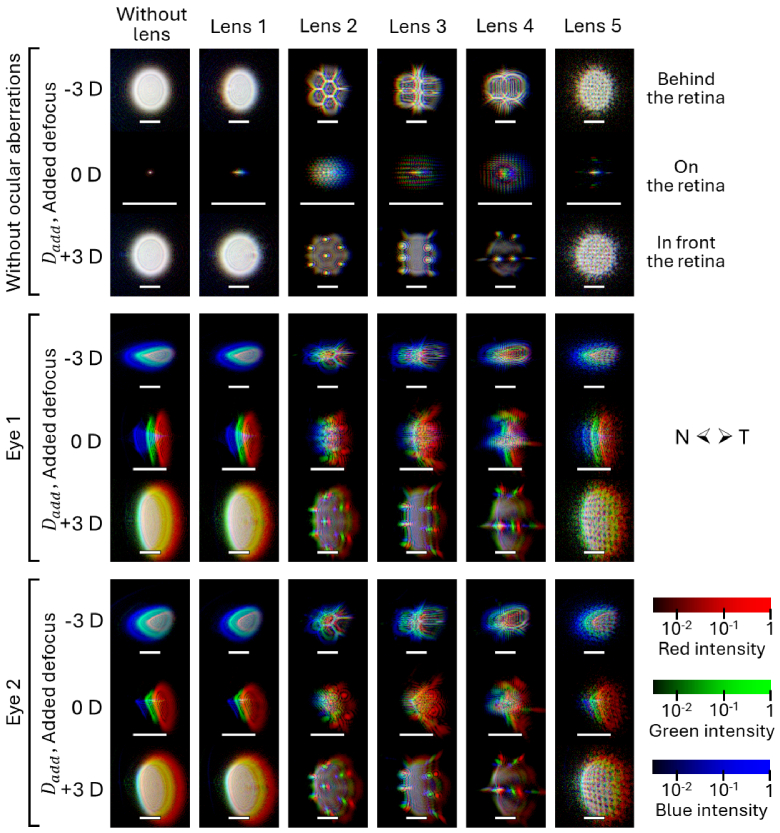
Overlay of monochromatic blue, green and red PSFs at the periphery (*ε*=30°). Each monochromatic PSF was normalized to its maximum. N and T denote the retinal nasal and temporal directions in each PSF. Scale bars, 20 minarc.

TCAs for each lens and eccentricity are listed in Table 2. Small differences between the centers of the pupil and lens led to nonzero values at *ε*=0°. Positive TCA means that the short wavelengths impinge the retina more toward the nasal retina than the longer wavelengths. As anticipated, higher-Abbe-number lenses (1 and 5) exhibited lower TCAs at the periphery than polycarbonate lenses. At the periphery, the TCA sign of the lenses is opposite to the calculated ocular TCA (8.0 and 10.6 minarc at *ε*=20° and 30°, respectively). Figure 4 shows the monochromatic PSFs at *ε*=30° (i.e., retinal temporal) overlaid and transversally shifted to visualize the chromatic aberrations of the lenses and the lens-eye systems, including the TCA. In this representation, each monochromatic PSF was normalized to its peak intensity. Without OAs, the effects of those aberrations are barely noticeable; however, those effects became easily recognizable on the in- and out-of-focus PSFs once the OAs were reproduced, leading to a lack of spatial coincidence of the three monochromatic patterns.

**Table 2.** Transverse chromatic aberration (TCA) in the tested lenses. The shown values correspond to the mean ± standard deviation (in minarc) after three measurements.

	Eccentricity		
	0°	20°	30°
Lens 1	-0.1 ± 0.1	-1.1 ± 0.1	-2.0 ± 0.2
Lens 2	0.4 ± 0.2	-2.1 ± 0.1	-3.5 ± 0.1
Lens 3	0 ± 0.3	-2.2 ± 0.2	-3.7 ± 0.4
Lens 4	0.2 ± 0.1	-2.4 ± 0.2	-3.7 ± 0.3
Lens 5	0.1 ± 0.1	-1.2 ± 0.3	-2.0 ± 0.1

**Fig. 5.**
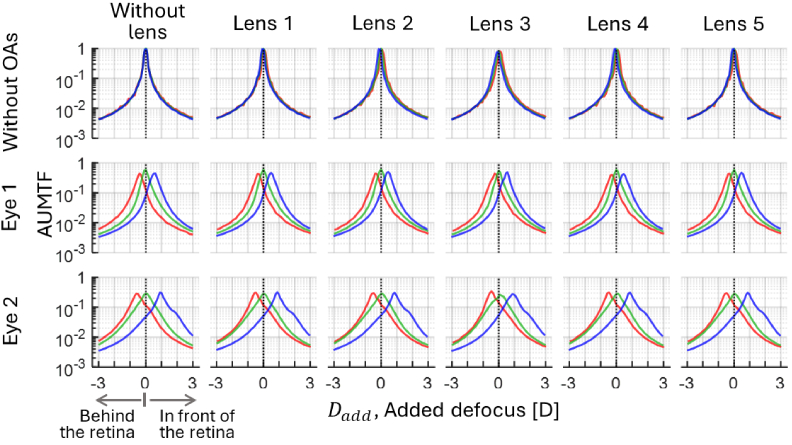
Through-focus AUMTFs of the instrument and the tested lenses, with and without adding ocular aberrations (OAs) through the central zone of the lenses (*ε*=0°). Positive and negative added defoci are associated to AUMTF values behind and in front of the retina, respectively. Red, green and blue lines depict the values for each spectral region.

**Fig. 6.**
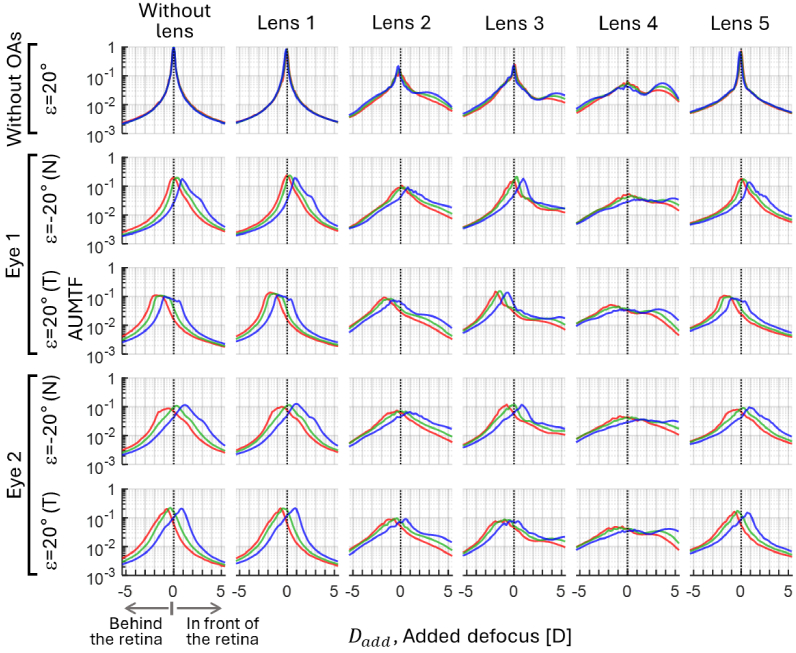
Through-focus AUMTFs of the instrument and the tested lenses, with and without adding ocular aberrations (OAs) at *ε*=±20°. Positive and negative added defoci are associated to AUMTF values behind and in front of the retina, respectively. Red, green and blue lines depict the values for each spectral region.

**Fig. 7.**
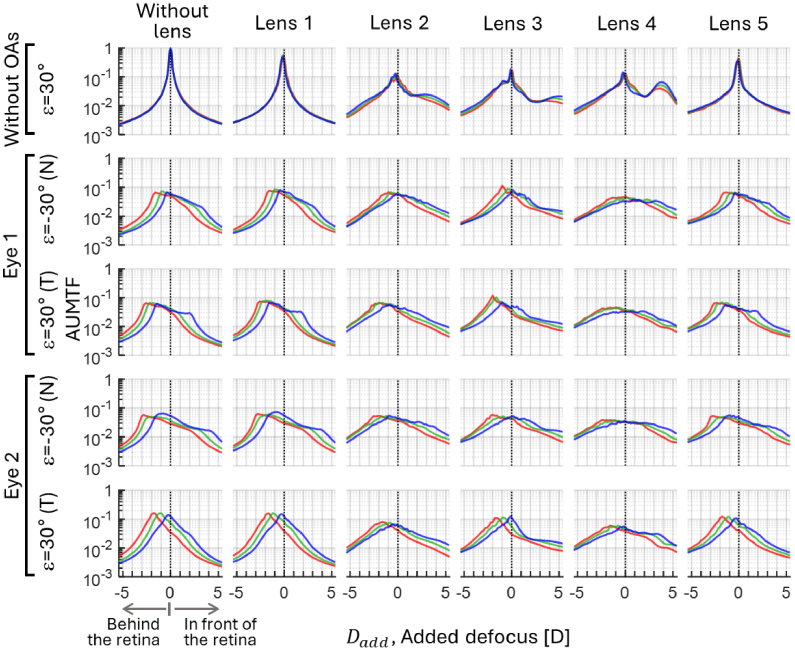
Through-focus AUMTFs of the instrument and the tested lenses, with and without adding ocular aberrations (OAs) at *ε*=±30°. Positive and negative added defoci are associated with AUMTF values behind and in front of the retina, respectively. Red, green and blue lines depict the values for each spectral region.

Figures 5, 6 and 7 show the through-focus AUMTFs (TF-AUMTFs) obtained with and without the tested lenses at eccentricities of 0°, ± 20° and ±30°, respectively. The optical response of the instrument (i.e., without OAs and without lens) was stable across the eccentricities, as demonstrated by the similarity among the TF-AUMTF values at different wavelengths. This stability proves that the following findings originate from the structures of the tested lens and OAs rather than the instrument.

The LCAs measured in each lens were similar across ε , and these values mainly depends on the material. The LCA for lenses 1 and 5, made from high-Abbe-number materials, was (mean ± std) -0.07 ± 0.01 D. In comparison, the average LCAs for the polycarbonate lenses were -0.12 ± 0.02 D for lens 2, -0.10 ± 0.02 D for lens 3, and -0.13 ± 0.02 D for lens 4. In contrast, the LCA for eyes 1 and 2 ranged from 0.91 to 1.16 D and from 1.50 to 1.93 D, respectively.

The peaks of AUMTF values are generally higher in blue than other spectral regions due to diffraction (i.e., diffraction patterns with the same defocus are less spread at shorter wavelengths). Without OAs, the dependence of the dioptric power of lenslets in lenses 2, 3, and 4 on λ is evident. Unlike lenses 3 and 4, lens 2 did not exhibit AUMTF peaks around the nominal power of its lenslets. The average power of lenslets (APL) in lenses 3 and 4 was estimated as the difference in defocus of the TF-AUMTF peaks around zero-defocus and within the myopic range. In lens 3, the APLs for [red, green and blue] were: [3.4, 4.1,4.5] D and [3.6, 4.5, 5.1] D at *ε*=20° and 30°, respectively. In lens 4, the APLs were [3.3, 3.5, 3.8] D and [3.6, 3.8, 4.0] D at *ε*=20° and 30°, respectively.

OAs did not significantly affect the AUMTF values at *ε*=0° since the central refractive errors were corrected. However, peripheral OAs reduce AUMFT for all lenses and concentrate the higher AUMTF values behind the retina.

**Fig. 8.**
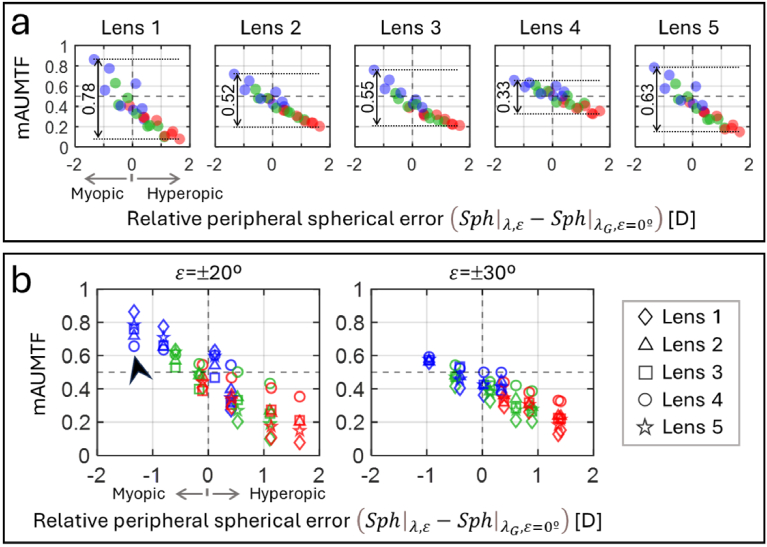
Integrated AUMTF values in front of the retina, normalized to the total AUMTF values on both sides of the retina (mAUMTF) as a function of the relative peripheral spherical error of the two reproduced eyes. The mAUMTF values are shown for: (a) each tested lens, and (b) each pair of tested eccentricities. Red, green and blue symbols depict the values for each spectral region.

Figure 8 shows the mAUMTF metric (see Eq. (2)), which quantifies the accumulated AUMTF values in front of the retina relative to the total values across both sides of the retina. For each wavelength, this metric is plotted as a function of the relative peripheral spherical error, defined as the difference between the peripheral spherical error and the central spherical error at $\lambda_{G}$ and calculated by using Eq. (3). Positive and negative relative peripheral spherical errors correspond to hyperopic and myopic errors, respectively.

Figure 8(a) simultaneously shows the mAUMTF at *ε*=±20° and ±30° for each lens. In general, mAUMTF values decrease with increasing relative peripheral spherical error, indicating that the distribution of AUMTF values on both sides of the retina is highly influenced by the eye's peripheral refraction and LCA. The peak-to-peak amplitude of the mAUMTF values across the relative peripheral spherical errors were 0.78, 0.52, 0.55, 0.33 and 0.63 for lenses 1 through 5, respectively. These amplitudes account for the dependence of the lenses’ performance on the peripheral spherical error of the eyes. Lens 5, based on diffusion optics technology, performed similarly to lens 1. Lenses 2, 3, and 4, which incorporated lenslet arrays, exhibited lower amplitudes due to a more uniform (or equalized) distribution of AUMTF values on both sides of the retina. Notably, lens 4 produced the lowest amplitude due to its higher fill factor (and larger lenslets at *ε*=±30°) compared to the other lenses.

Figure 8(b) compares the mAUMTF values among the tested lenses for each pair of eccentricities (i.e., ± 20° and ±30°), providing a complementary analysis. The difference between the maximum and minimum mAUMTF values at single relative spherical errors is higher for *ε*=±20° than for *ε*=±30°. This disparity is due to the increased astigmatism and high-order aberrations with eccentricity, which flatten the through-focus AUMTFs. Moreover, the equalization effect of the lenslets (previously discussed) is evident when comparing the performance of the lens 4 with respect to lens 1 across all eccentricities. In the hyperopic range of peripheral spherical errors, lens 1 produced the lowest mAUMTF values, while lens 4 achieved the highest values. However, in the myopic range, particularly for the blue illumination, this performance trend was reversed (as marked by the black arrow).

## 4. Discussion

The chromatic imaging properties of MC spectacle lenses were measured using an instrument based on spatial light modulation technology. The PSFs were acquired at three different wavelengths (red, green and blue) while correcting the chromatic errors from the instrument itself and reproducing the aberrations of two myopic eyes. To the best of our knowledge, this is the first study on the chromatic characterization of MC spectacle lenses and the first characterization of lens 4. Lenses 2, 3 and 5 were previously tested under monochromatic illumination [4–8].

The chromatic characterization of the MC lenses initially showed differences in TCA and LCA that can be attributed to their different materials. The TCA signs of the eye and lens differ, indicating a partial compensation for this type of aberration. However, the spatial coincidence of the peripheral PSFs at different wavelengths on the retinal plane of the lens wearer is unlikely, mainly due to LCA and diffraction effects (see Fig. 4). Additionally, the analysis of the TF-AUTMTF profiles of lenses 2, 3 and 4 without OAs (see Figs. 6 and 7) revealed that the LCA of their lenslets shares the sign with the ocular LCA. For instance, lenslets in lens 3 focused blue light between 1.1 and 1.5 D in front of the red focus, while in lens 4, the blue focus was between 0.4 and 0.5 D in front of the red focus. Moreover, the diffraction of light in the lenslets led to a wavelength-dependent spatial concentration of light in their focal spots. For example, the lenslets in lens 3 led to an AUMTF peak at their blue focus between 1.4 and 1.5 times higher than the peak at red. Likewise, in lens 4, the AUMTF peak generated by its lenslets at blue was 1.6 to 1.8 times higher than the corresponding peak at red.

The interaction between the MC lenses and the OAs was also investigated. Peripheral OAs induced focal shifts and spread the AUMTF values. These effects were assessed through the mAUMTF metric, which quantified the total AUMTF values in front of the retina relative to the total AUMTF values on both sides of the retina. The mAUMTF depended on the eye's relative peripheral spherical defocus error, as illustrated in Fig. 8(b). The hyperopic spherical defocus errors (mostly characteristic of longer wavelengths) reduce mAUMTF values as the focal plane shifts behind the retina, while the myopic defocus errors increase mAUMTF values. This dependence strongly influences the performance of lenses 1 (a single-vision lens) and 5. The found similarity in the performance of these lenses reinforces a prior observation [7], in which the contrast reduction of the images at the retina expected for lens 5 proves more efficient under photopic lighting conditions. On the other hand, lenses with incorporated lenslets appear to equalize mAUMTF values across the range of relative peripheral spherical defocus errors (see Fig. 8(a)), redeploying the AUMTF values around the retina. Lenses 2 and 3, which proved similar clinical efficacy in slowing myopia progression [21], led to similar mAUMTF values despite the differences in their lenslet array (see Table 1). Lens 4 led to the strongest equalization effect, likely due to its higher fill factor and increasing size of their larger with the eccentricity.

The chromatic characterization was also analyzed to provide insights into the design of future MC lenses, considering that in myopic eyes, unlike emmetropic and hyperopic eyes, the relative peripheral spherical defocus errors tend to be more hyperopic [22,23]. According to the simultaneous competing blur theory, the projection of sharp stimuli on or in front of the peripheral retina can trigger a slowing or reversal of eye elongation [17]. This theory supported the design of the tested MC lenses with incorporated lenslets. However, none of these lenses effectively repositioned red images in front of the retina and compensated for the effects of the ocular peripheral refraction at the red wavelength. As shown in Fig. 8(a), most of the red mAUMTF values for lenses 2, 3 and 4 were lower than 0.5, indicating that the red stimuli were predominantly imaged behind the retina. This deficiency could be due to the lower dioptric power of the lenslets in red compared to blue. Hence, although the photopic spectral sensitivity of the human eye is maximized at 555 nm [24], and this wavelength is commonly used to design ophthalmic lenses, the design of the peripheral structures of MC lenses should optimize their imaging capabilities at longer wavelengths (e.g., red or near-infrared). This design approach ensures projecting sharp images in front of the peripheral retina for both long and short wavelengths. Furthermore, the development of customized MC lenses, which potentially improves the efficacy of myopia progression treatments [3], could be guided by peripheral refraction errors measured under red or near-infrared illumination. Using long-wavelength light for these measurements offers several benefits: it improves patient comfort, increases retinal reflectivity, and provides higher safety thresholds for retinal light exposure, particularly in the near-infrared spectrum [25].

In this study, OAs of two myopic eyes with similar central refractions but differing profiles of peripheral refractions and high-order aberrations were analyzed. These cases highlight the necessity of designing optical treatments for myopia progression based on the ocular wide-angle OAs rather than relying solely on the central refraction or applying a standardized design across a broad myopic population. The reproduced OAs are part of a dataset collected from a small population of adult eyes [20]. To our knowledge, no studies report children’s wide-angle OAs measured across different visible wavelengths. It is important to remember that the MC strategies are primarily targeted at subjects in their middle childhood when the eye is still undergoing growth. However, despite the limited number of eyes analyzed in this study and the adult age range of the subjects, the insights gained for developing future MC lenses remain valid. This assertion is supported by the observation that the eye and lenslets exhibit LCAs with the same sign, where blue light focuses in front of red light. Consequently, designing lenslets to create a red image in front of the retina would unavoidably position the blue image in front of the retina. Including additional myopic eyes in future studies would provide more comprehensive data to evaluate the effect of the entire set of OAs, not only defocus (as shown in Fig. 8), on the mAUMTF values.

Our study has several limitations. First, the maximum amplitude of the added defocus was ±5.4 D, being limited by the size of the camera sensor. Second, the pupil size in this study (4 mm) was smaller than that typically found in children (the population targeted for MC), as the adopted aberrometric measurements conditioned it. Third, the pixelated structure of the SLM generates PSF replicas on the image plane, which could overlap if they are wider than 2.4°. Lenses incorporating diffusers with strong scattering may produce such overlap. However, this was not the case of lens 5, which produces scattered light at angles greater than 1° with intensities several orders of magnitude lower than the central peak [7]. Alternative methodologies (e.g., optical integration method [26]) may be more suitable to investigate the imaging properties of scattering-based lenses. Fourth, only a single aperture on the MC lens was selected and tested for each eccentricity. Since the periods of the peripheral arrays at the tested eccentricities were smaller than 4 mm (the pupil size), the structures fit within the pupil and were evaluated, as depicted in Fig. 1. However, selecting multiple apertures at a single eccentricity would enable the determination of tolerance values for the calculated image quality metrics. Last, the TCA effects on the image quality at the eye fundus cannot be accurately quantified since the TCAs were not measured in the reproduced eyes. Instead, ocular TCAs were calculated for visualization of the chromatic PSFs only.

In conclusion, we characterized the chromatic imaging properties of MC spectacle lenses three eccentricities along the horizontal meridian using liquid crystal technology. As part of this characterization, we quantified the ability of the lenslets to generate sharp images in front of the peripheral retina, which is the primary purpose of their incorporation, while reproducing the effects of the OAs of two myopic eyes. Our findings indicate that, in general, MC lenses were not effective at substantially improving the quality of red images focused in front of the retina. However, the lenslet arrays with a higher fill factor and larger lenslets performed the best in increasing the quality of those images. Additionally, it was found that the lenslets exhibited LCAs of the same sign as the eye, focusing blue light in front of red light. Therefore, designing lenslets or other optical structures to generate sharp stimuli in front of the retina with long wavelengths (e.g., red) will also produce stimuli in front of the retina with shorter wavelengths (e.g., blue). This premise suggests that using red or infrared aberrometry and refractometry data is suitable for guiding the development of future optical interventions for MC.

## Supplemental information

Supplement 1 Figure S1 and Table S1 https://doi.org/10.6084/m9.figshare.28449146

## Data Availability

Data underlying the results presented in this paper are not publicly available at this time but may be obtained from the authors upon reasonable request.

## Supplemental document

See Supplement 1 for supporting content.
