# Supplementary material for: Chromatic imaging properties of myopia control spectacle lenses
Source: Biomed Opt Express. 2025 Mar 19;16(4):1499–512. doi: 10.1364/BOE.545932 (PMC12047712; doi:10.1364/BOE.545932)
Supplement: Supplementary file 1 [file boe-16-4-1499-s001.pdf]

Supplemental Document

Biomedical Optics EXPRESS

Chromatic imaging properties of myopia control spectacle lenses: supplement

AUGUSTO ARIAS,[1,*] SUSANNA P. CLEMENT,[1] PABLO ARTAL,[2] AND SIEGFRIED WAHL[1,3]

[1] *ZEISS Vision Science Lab, Ophthalmic Research Institute, University of Tübingen, Tübingen, Germany*
[2] *Laboratorio de Óptica, Universidad de Murcia, Campus de Espinardo, Murcia 30100, Spain*
[3] *Carl Zeiss Vision International GmbH, Technology & Innovation, Aalen, Germany*
[*] *augusto.arias-gallego@uni-tuebingen.de*

This supplement published with Optica Publishing Group on 19 March 2025 by The Authors under the terms of the Creative Commons Attribution 4.0 License in the format provided by the authors and unedited. Further distribution of this work must maintain attribution to the author(s) and the published article's title, journal citation, and DOI.

Supplement DOI: https://doi.org/10.6084/m9.figshare.28449146

Parent Article DOI: https://doi.org/10.1364/BOE.545932

Chromatic imaging properties of myopia-control spectacle lenses: supplemental document

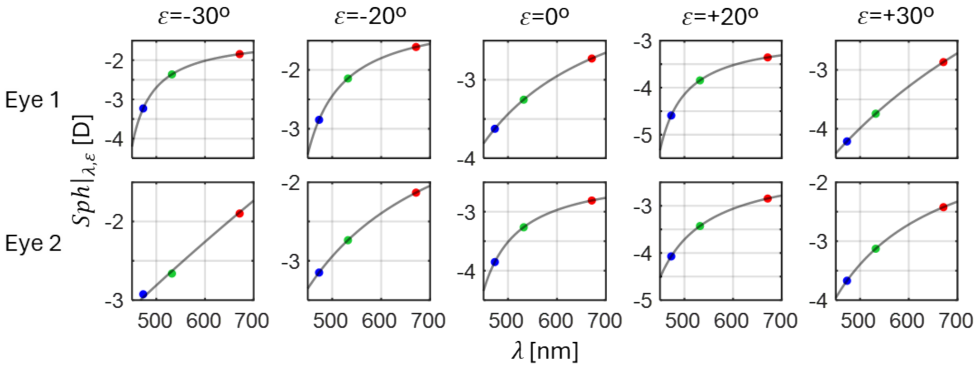

Fig. S1. Spherical error ($Sph|_{\lambda,\varepsilon}$) as a function of the wavelength λ, which is fitted with Cornu's hyperbolic formula Γ (gray continuous line), for the two reproduced eyes.

Table S1. Parameters of Cornu's hyperbolic formula across the tested eccentricities for the two reproduced eyes.

		**Eccentricity**				
**Parameter**		-30º	-20º	0	+20º	+30º
p	Eye 1	-1.376	-1.018	-1.386	-2.881	2.263
	Eye 2	17.486	-0.449	-2.305	-2.079	-0.895
q	Eye 1	124.0	172.9	664.1	130.4	4.879×10^{3}
	Eye 2	4.879×10^{4}	885.3	148.5	247.0	673.3
g	Eye 1	406.1	378.5	176.3	396.7	-280.4
	Eye 2	-3.068×10^{3}	145.1	377.1	349.1	230.4
